# Molecular characterization and epidemiological aspects of non-polio enteroviruses isolated from acute flaccid paralysis in Brazil: a historical series (2005–2017)

**DOI:** 10.1080/22221751.2020.1850181

**Published:** 2020-12-01

**Authors:** Ivanildo P. Sousa, Maria de Lourdes Aguiar Oliveira, Fernanda M. Burlandy, Raiana S. Machado, Silas S. Oliveira, Fernando N. Tavares, Francisco Gomes-Neto, Eliane V. da Costa, Edson E. da Silva

**Affiliations:** a Laboratório de Enterovírus, Instituto Oswaldo Cruz, Fundação Oswaldo Cruz, Rio de Janeiro, Brazil; b Laboratorio de Vírus Respiratórios e do Sarampo, Instituto Oswaldo Cruz, Fundação Oswaldo Cruz, Rio de Janeiro, Brazil; c Laboratório de Referência Regional em Enteroviroses, Seção de Virologia, Instituto Evandro Chagas, Ananindeua, Brazil; d Laboratório de Toxinologia, Instituto Oswaldo Cruz, Fundação Oswaldo Cruz, Rio de Janeiro, Brazil

**Keywords:** Non-polio enterovirus, acute flaccid paralysis, enterovirus, molecular epidemiology, poliomyelitis surveillance

## Abstract

Due to the advanced stage of polio eradication, the possible role of non-polio enteroviruses (NPEVs) associated to acute flaccid paralysis (AFP) cases has been highlighted. In this study, we described epidemiological aspects of NPEVs infections associated to AFP and explore the viral genetic diversity, information still scarce in Brazil. From 2005 to 2017, 6707 stool samples were collected in the scope of the Brazilian Poliomyelitis Surveillance Program. NPEVs were isolated in 359 samples (5.3%) and 341 (94.9%) were genotyped. About 46 different NPEV types were identified with the following detection pattern EV-B > EV-A > EV-C. The major EV-types were CVA2, CV4, EV-A71, CVB3, CVB5, E6, E7, E11, CVA13 and EV-C99, which corresponds to 51.6% of the total. Uncommon types, such as CVA12, EV-90 and CVA11, were also identified. Different E6 genogroups were observed, prevailing the GenIII, despite periods of co-circulation, and replacement of genogroups along time. CVA2 sequences were classified as genotype C and data suggested its dispersion in South-American countries. CVA13 viruses belonged to cluster B and Venezuelan viruses composed a new putative cluster. This study provides extensive information on enterovirus diversity associated with AFP, reinforcing the need of tailoring current surveillance strategies to timely monitor emergence/re-emergence of NPEVs.

## Introduction

Worldwide, enteroviruses (EVs) are one of the major causes of human viral infections. EVs (genus *Enterovirus*, family *Picornaviridae*) are small, non-enveloped, positive-sense, single-stranded RNA viruses. The genomic RNA (∼7.5 kb), encodes a large polyprotein, further processed into mature structural (VP1 to VP4) and nonstructural proteins [1]. EVs are classified into fifteen species: EV A-L and human rhinoviruses (HRV A-C) [2]. Seven species (EV A-D and HRV A-C) are able to cause human infection [3]. These viruses present a high genetic diversity, due to cumulative mutations and/or recombinations [4,5]. These events are frequently observed between polioviruses and other species C enteroviruses [4,6], favouring the emergence of novel viruses of unpredictable pathogenicity and clinically unknown outcomes [7].

EVs are responsible for a wide variety of clinical disease, ranging from common cold to cutaneous syndromes, visceral and neurological disorders, as acute flaccid paralysis (AFP) [8]. AFP is a clinical disorder with a broad array of etiologies, including infectious and non-infectious causes [9,10]. Polioviruses are the main cause of AFP, however, a considerable proportion of non-polio enteroviruses (NPEVs) has been increasingly associated with this neurological disorder [11].

AFP surveillance is part of a global programme coordinated by the World Health Organization (WHO) and conducted by the Brazilian Ministry of Health (MoH) at the national level. These activities have been kept at appropriate levels in Brazil [12]. In the context of global eradication of polioviruses, information on NPEVs circulation is key to understand their role in AFP context. Surveillance data shows that in Europe, USA and Africa, most cases have been associated with EV-B species, whereas species A are normally more prevalent in Asia [13–17]. The etiology of AFP cases in Brazil is systematically determined, although information on molecular diversity and NPEV circulation patterns is still scarce [18].

In this report, we assessed the prevalence of different EVs among AFP cases and explored the molecular diversity and putative circulation patterns of CVA2, E6 and CVA13 viruses in different Brazilian geographical regions, along 13 years of surveillance (2005–2017). Our findings highlight a high genomic diversity of EV strains, reinforcing the need to improve the current surveillance programmes, to regularly detect and characterize at molecular level the circulating viral strains.

## Methods

### Ethics statements

Samples were collected in the scope of the Global Enterovirus Surveillance Program, as part of the WHO and National Public Health responses, dispensing a formal review by IRB.

### Specimens and virus isolation

Stool samples from 6707 AFP symptomatic patients were collected by the Brazilian National Public Health network (2005–2017). These specimens are routinely sent to our laboratory, a WHO Regional Reference Laboratory and National Reference Laboratory/MoH. Samples were processed according to the WHO standard procedures [19]. A chloroform-treated stool suspension (200 µL) was inoculated into RD (human rhabdosarcoma), HEp-2C (human epidermoid carcinoma) and L20B (cell line expressing poliovirus receptor) cell lines as previously described [12,19]. Cell cultures showing cytopathic effect (CPE) were harvested and kept (−20°C) until typing. EV molecular detection was performed by conventional RT–PCR in the culture supernatant. Positivity was defined as the presence of CPE and EV positive result in RT–PCR.

### Molecular detection and sequencing

Viral RNA was extracted from culture supernatant using the QIAamp viral RNA mini kit (Qiagen, Hilden, Germany). RT–PCR was carried out using the primer pairs 292/222 and 222/224 (for partial VP1) and 224/011 (for entire VP1) [12,19]. For amplification, 3ul of cDNA was added to RT–PCR mix (25 µL Master Mix, 20 µL H_2_O and 1 µL of each primer). After 32 cycles (94°C/30s, 42°C/30s, and 60°C/2 min), PCR products were submitted to electrophoresis, amplicons were gel-purified (QIAquick Gel Extraction Kit, Qiagen) and cycle-sequenced (ABI PRISM BigDye Terminator v.3.1, ABI, USA).

### Phylogenetic analysis

E6, CVA2, CVA13, E7, EV-A71 and CVB5 VP1 sequences were deposited at the GenBank (NCBI), under the accession numbers MT212610 to MT212635 (E6 and CVA2), MT271232 to MT271239 (CVA13) and MT937026 to MT937062 (E7, CVB5 and EV-A71). For phylogenetic reconstruction, representative sequences were selected according to nucleotide similarity (BLAST). For E6, CVA2, CVA13, E7, CVA2 and EV-A71 the sequences D’Amori (AY302558.1), Fleetwood (AY421760.1), Flores (AF499637.1), Wallace (AF465516.1), Faulkner (AF114383.1) and BrCr (U22511) were used as reference strains, respectively. Reference sequences for viral genogroups, genotypes and/or clusters were also included [17,20–22]. VP1 sequences were edited using Bioedit (7.2.5.0)/SeqMan (7.0.0) programmes and aligned by Muscle [23]. Phylogenetic trees were reconstructed using a Maximum Likelihood algorithm (PhyML) [24], with a GTR + I+G nucleotide substitution model, as estimated in JModeltest v2.1.7 [25]. The phylogenetic trees were edited using Figtree 1.4.3.

## Results

### NPEV isolation and distribution

**Figure 1.**
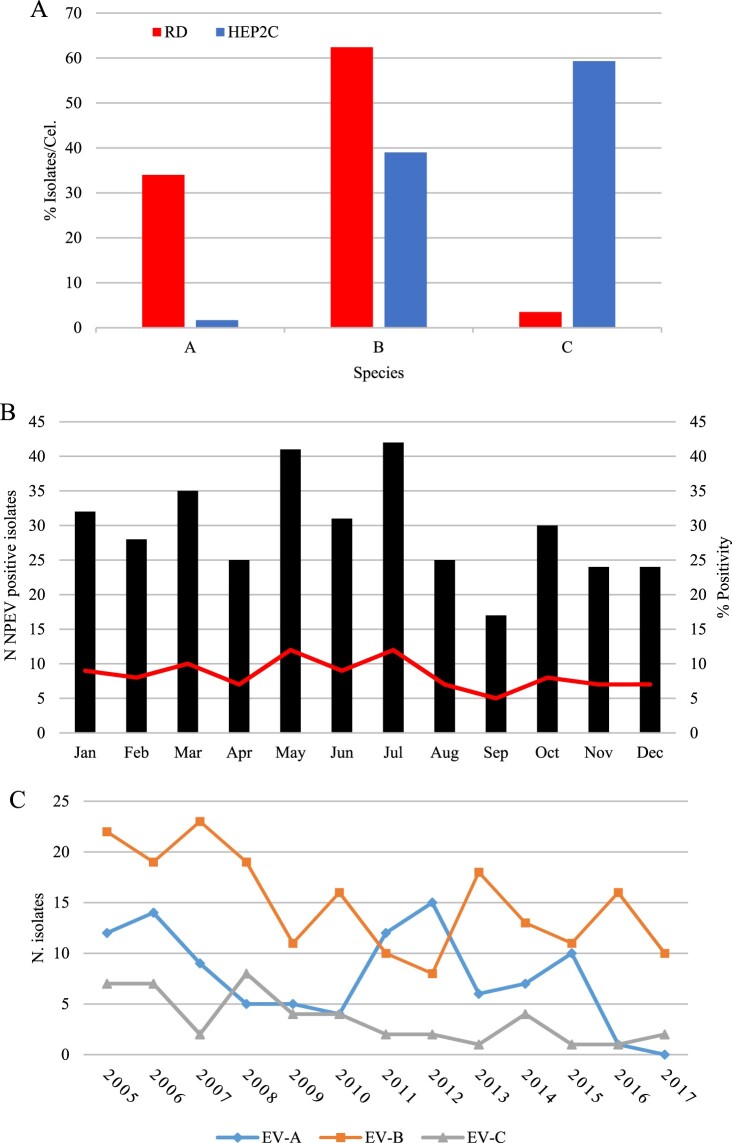
Isolation of non-polio enterovirus and seasonal distribution from AFP cases in Brazil from 2005 to 2017. **(A)** Relative isolation from RD (red bars) or Hep2c (blue bars) cells. **(B)** Proportion of NPEV detection based on cell culture isolation and monthly distribution. **(C)** Annual distribution of NPEV isolates, according to EV species.

NPEV was isolated from 359 samples (359/6707; 5.3%), in RD or HEp-2C cell culture, but remained negative in L20B cells. Of these 359, 213 (59.3%) were successfully isolated in RD cells, 54 (15.1%) in HEp-2C and 92 (25.6%) in both cells lines (Table 1). Among positive samples, 341 were successfully typed (RD, 282 and HEp-2C, 59). EV-A (96/282; 34%) and EV-B (176/282; 62.4%) species were isolated mainly in RD cells, while EV-C (35/59; 59.3%) species was detected mostly in HEp-2C (Figure 1(A)). No clear seasonal distribution of NPEV infections could be identified within the investigated period. Despite a putative lower incidence in September, the relative frequencies of viral infections per month (5–12%; mean, 8.4%) suggested a uniform distribution throughout the year (Figure 1(B)).

**Table 1.** Frequency of cytopathic effect, according to cell line for enteroviruses.

Cytopathic effect per cell line			
Year	RD	HEp-2C	RD/HEp-2C
**2005**	30 (69.8)	5 (11.6)	8 (18.6)
**2006**	24 (57.1)	11 (26.2)	7 (16.7)
**2007**	27 (77.1)	1 (2.9)	7 (20)
**2008**	16 (44.5)	8 (22.2)	12 (33.3)
**2009**	9 (45)	6 (30)	5 (25)
**2010**	9 (37.5)	3 (12.5)	12 (50)
**2011**	18 (64.3)	5 (17.8)	5 (17.9)
**2012**	14 (56)	2 (8)	9 (36)
**2013**	13 (48.1)	5 (18.5)	9 (33.4)
**2014**	12 (50)	4 (16.7)	8 (33.3)
**2015**	18 (75)	0	6 (25)
**2016**	14 (73.7)	1 (5.3)	4 (21.1)
**2017**	9 (75)	3 (25)	0
**Total**	213	54	92

Note: Number between brackets mean percentage.

### Molecular typing of NPEV isolates

From 359 samples, 341 (94.9%) were classified into 46 different EV types and 18 remained untypeable (5.1%) (Table 2). Among the 341 typed isolates, 100 were classified into 13 types of EV-A (29.3%), 196 into 27 types of EV-B (57.5%) and 45 into 6 types of EV-C species (13.2%). No EV-D species was found. The ten most common types (CVA2, CVA4, EV-A71, CVB3, CVB5, E6, E7, E11, CVA13 and EV-C99) accounted for about half of samples (51.6%). CVA2 (16/341; 4.7%) and EV-A71 (26/341; 7.6%) were the most frequent types within EV-A; E6 (26/341, 7.3%) and E11 (23/341, 6.7%) among EV-B, whereas CVA13 (17/341, 5.0%) and EV-C99 (13/341, 3.8%) among EV-C species isolates. Nine types (CVA12, EV-A90, CVB2, E1, E2, E9, E15, E27 and CVA17) were detected only once. Except for 2011–2012 – when EV-A species was predominant – EV-B was the most prevalent specie (Figure 1(C)). Moreover, EV-C species accounted for 17.5% (7/40) and 25% (8/32) of annual isolation in 2006 and 2008, respectively (Figure 1(C)).

**Table 2.** Frequency of NPEV type distribution among AFP cases, Brazil 2005–2017.

Species	Type	Isolation year													N. total (% of total)
		05	06	07	08	09	10	11	12	13	14	15	16	17	
A	CVA2	2	2	1	1	1	1	1	5	2	0	0	0	0	16 (4.7)
	CVA3	2	0	0	0	0	0	0	0	0	0	0	0	0	2(0.6)
	CVA4	0	1	1	0	2	0	3	1	0	2	3	0	0	13 (3.8)
	CVA5	0	3	0	2	0	1	2	0	0	0	0	0	0	8 (2.3)
	CVA6	1	0	0	0	2	0	0	0	0	0	0	0	0	3 (0.9)
	CVA7	1	0	0	1	0	0	0	0	0	0	0	0	0	2 (0.6)
	CVA8	1	0	1	0	0	0	0	3	0	0	1	0	0	6 (1.8)
	CVA10	0	3	1	0	0	0	4	0	0	0	0	0	0	8 (2.3)
	CVA12	0	0	0	0	0	0	0	1	0	0	0	0	0	1 (0.3)
	CVA14	1	0	2	0	0	0	0	0	0	0	0	0	0	3 (0.9)
	CVA16	2	1	0	0	0	1	1	1	2	1	2	0	0	11 (3.2)
	EV-A71	2	3	3	1	0	1	1	4	2	4	4	1	0	26 (7.6)
	EV-A90	0	1	0	0	0	0	0	0	0	0	0	0	0	1 (0.3)
Subtotal		12	14	9	5	5	4	12	15	6	7	10	1	0	100 (29.3)
B	CVB1	0	2	0	0	1	1	0	0	1	0	0	0	0	5 (1.5)
	CVB2	2	1	0	0	0	1	3	0	0	1	0	2	1	11 (3.2)
	CVB3	1	0	1	0	2	3	2	0	1	0	2	1	0	13 (3.8)
	CVB4	3	0	0	2	1	1	0	0	2	1	0	2	0	12 (3.5)
	CVB5	1	1	2	0	0	1	0	1	4	1	1	1	1	14 (4.1)
	E1	0	0	0	0	0	0	0	0	0	0	0	1	0	1 (0.3)
	E2	0	0	1	0	0	0	0	0	0	0	0	0	0	1 (0.3)
	E3	2	1	0	2	1	0	0	1	0	0	0	0	1	8 (2.3)
	E4	0	1	0	1	1	0	0	0	0	0	0	0	0	3 (0.9)
	E6	0	3	6	3	1	3	1	0	2	2	2	1	1	25 (7.3)
	E7	1	1	3	2	0	1	0	0	2	3	0	1	2	16 (4.7)
	E9	0	1	0	0	0	0	0	0	0	0	0	0	0	1 (0.3)
	E11	0	1	3	4	2	3	2	1	2	2	3	0	0	23 (6.7)
	E13	0	3	2	2	0	1	1	1	0	0	0	0	0	10 (2.9)
	E14	0	0	1	0	0	0	1	2	1	1	0	1	0	7 (2.1)
	E15	0	0	0	0	0	0	0	0	0	0	1	0	0	1 (0.3)
	E16	1	0	0	0	0	0	0	0	0	0	0	1	0	2 (0.6)
	E18	0	0	1	1	0	0	0	0	0	1	0	1	1	5 (1.5)
	E19	1	0	0	1	0	1	0	0	0	0	0	0	0	3 (0.9)
	E20	1	0	0	1	1	0	0	0	0	1	0	1	1	6 (1.8)
	E21	1	0	0	0	0	0	0	1	1	0	1	0	1	5 (1.5)
	E24	2	1	0	0	0	0	0	1	0	0	0	0	0	4 (1.2)
	E25	1	1	0	0	1	0	0	0	1	0	1	0	0	5 (1.5)
	E27	1	0	0	0	0	0	0	0	0	0	0	0	0	1 (0.3)
	E29	0	0	2	0	0	0	0	0	0	0	0	1	0	3 (0.9)
	E30	2	0	1	0	0	0	0	0	1	0	0	2	1	7 (2.1)
	E33	2	2	0	0	0	0	0	0	0	0	0	0	0	4 (1.2)
Subtotal		22	19	23	19	11	16	10	8	18	13	11	16	10	196 (57.5)
	CV-A11	0	1	0	1	0	0	0	0	0	0	0	0	0	2 (0.6)
C	CV-A13	2	4	2	3	1	0	0	2	1	0	0	1	1	17 (5)
	CV-A17	1	0	0	0	0	0	0	0	0	0	0	0	0	1 (0.3)
	CV-A21	0	0	0	0	0	0	0	0	0	2	0	0	0	2 (0.6)
	CV-A24	3	0	0	2	2	0	2	0	0	0	0	0	1	10 (2.9)
	EV-C99	1	2	0	2	1	4	0	0	0	2	1	0	0	13 (3.8)
Subtotal		7	7	2	8	4	4	2	2	1	4	1	1	2	45 (13.2)
Total		41	40	34	32	20	24	24	25	25	24	22	18	12	341 (100)

Our data suggest a temporal change in the distribution of NPEVs species. In 2007, E6 accounted for 17.6% (6/34) of annual isolation, but none detection occurred in 2005 and 2012. CVA2 was rare before 2012, when accounted for 20% of viral infections, disappearing afterwards (2014–2017) (Table 2).

### Phylogenetic analyses

**Figure 2.**
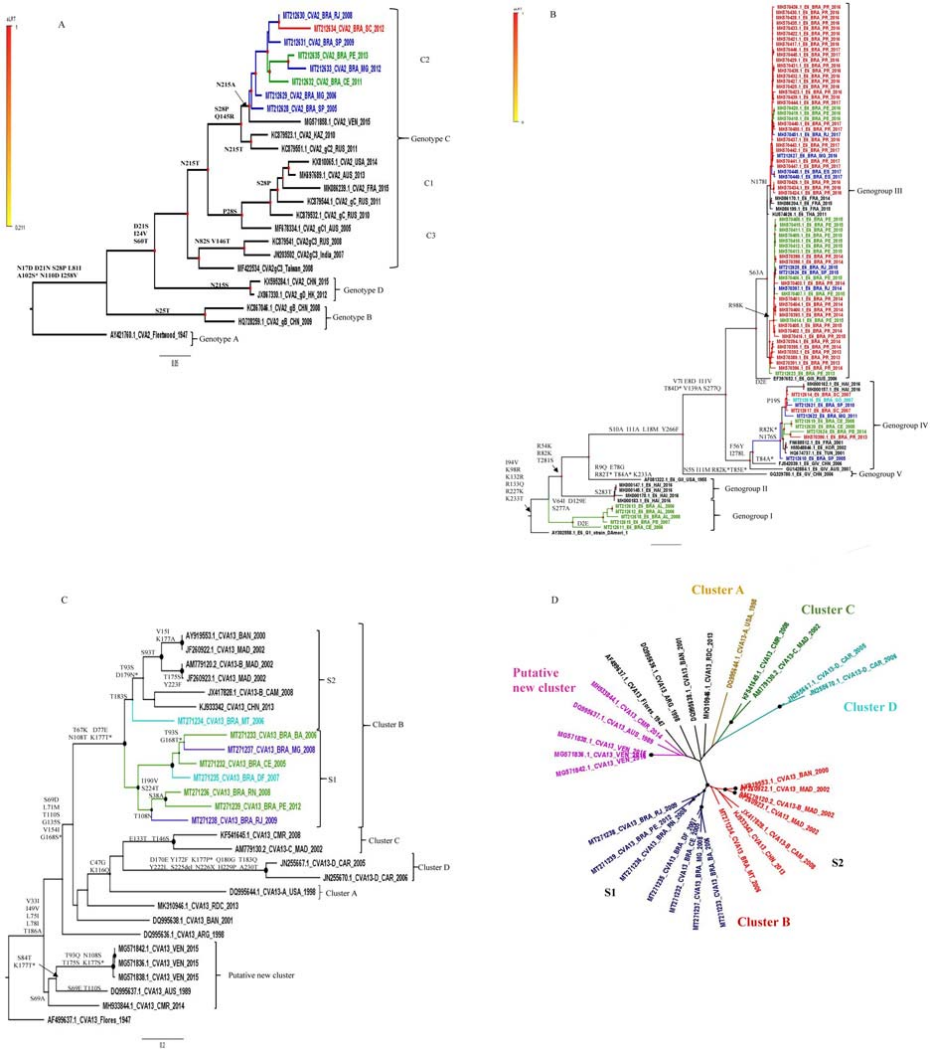
Maximum likelihood phylogenetic trees of Brazilian and global representative NPEV based on CVA2 (869 bp) **(A)**, E6 (851 bp) **(B)** and CVA13 (707 bp) **(C,D)** VP1 sequences. Sequences were coloured according to the Brazilian geographical region and the main aminoacid substitutions are shown. Substitutions between aminoacid correspondent to the BC loop are indicated by an asterisk (*). The strains of this study are identified by the GenBank accession number (MT212610 to MT212635, E6 and CVA2 and MT271232 to MT 271239, CVA13). The Brazilian states were identified as Northeast (green: AL, Alagoas; PB, Paraíba, PE, Pernambuco; BA, Bahia; CE, Ceará; RN, Rio Grande do Norte), Southeast (blue: ES, Espírito Santo; MG, Minas Gerais; RJ, Rio de Janeiro; SP, São Paulo), Midwest (light blue: DF, Distrito Federal; MT, Mato Grosso) and South (red: SC, Santa Catarina).

CVA2, E6 and CVA13 reconstructed phylogenetic trees are shown in Figure 2. Brazilian CVA2 sequences (2005–2013) grouped with a representative from Venezuela (MG571858.1) into a single genetic cluster of genotype C (subgenotype C2) (Figure 2(A)). Subgenotype C1 included representatives from different continents (2005–2015), suggesting a global distribution.

E6 Brazilian sequences were distributed into three distinct genogroups (Figure 2(B)). The genogroup I (reference AY302658.1) was composed of 2 clusters. The Brazilian cluster – defined by substitutions V64I, D129E and S277A – included former Northeastern isolates (2006–2008). The genogroup IV – represented by FJ542039.1 and defined by the substitutions F56Y and I278L – circulated in different countries since the early 2000s and included all Brazilian regions (2007–2014). Additionally, the majority of sequences identified in this study were classified inside this group. GIII comprised the most recent isolates (2013–2017) subdivided into two major Brazilian clusters, characterized by substitutions R98K (2013–2015) and N178I (2016–2017), respectively. The co-circulation of different E6 genogroups was observed in determined years. Nevertheless, our data suggests the replacement of one genogroup by another along time, prevailing the circulation of type III in the last years.

The phylogenetic analyses of CVA13 revealed that Brazilian viruses belonged to cluster B, defined by the substitutions T67K, D77E, N108T and K177T (Figure 2(C)). Cluster B comprised two distinct clades S1 and S2. Out of one, our sequences were grouped into S1. The MT271234 sequence was into S2 clade, showing a weak statistical support. The nucleotide similarity within and between S1 and S2 was 81.1% vs. 83.3% and 78.4%, respectively (Table 3). Of note, Venezuelan sequences (MG571836.1, MG571838.1, MG571842.1) were grouped into a putative new CVA13 cluster (non A-F), defined by the substitution S69A. Sequences from this clade showed 83.2% of within nucleotide similarity, whereas these figures ranged from 72.5% to 75.8%, when compared to other CVA13 clusters (A-D) (Table 3). Altogether, these outcomes are compatible with a new putative CVA13 cluster circulating in South America since 2015, at least. Three CVA13 sequences and the prototype strain (AF499637.1) remained without specific clustering (Figure 2(D)).

**Table 3:** Nucleotide similarity with CVA13 clusters.

	N	(%)
Within CVA13 clusters	** **	** **
*Cluster A*	1	
*Cluster B*	14	80.2
* Clade S1*	7	81.1
* Clade S2*	7	83.3
*Cluster C*	2	78.3
*Cluster D*	2	83.2
*Putative new cluster*	5	83.2
Between CVA13 clusters		
*Between A, B, C, D*		70.9–73.5
*New cluster compared to clusters A-D*		72.5–75.8

The phylogenetic analysis based on VP1 sequences was also performed for the other highly frequent NPEVs, such as EV-A71, E7 and CVB5 (supplementary Figure S1). Although EV-A71 was the most prevalent within EV-A species viruses, only a small fragment (300 bp) was available for the majority of Brazilian sequences. The most recent Brazilian sequences could be classified as genotype C2 (2015–2016), whereas those obtained in former years belonged to genotype B2 (1999–2014). EV-A71, E7 and CVB5 revealed a genetic diversity. CVB5 sequences were classified as genogroup C, together with European and USA representatives. E7 Brazilian sequences were grouped into different clusters, including global representatives from distinct countries (supplementary Figure S1).

### Amino acid substitutions in the BC loop

**Figure 3.**
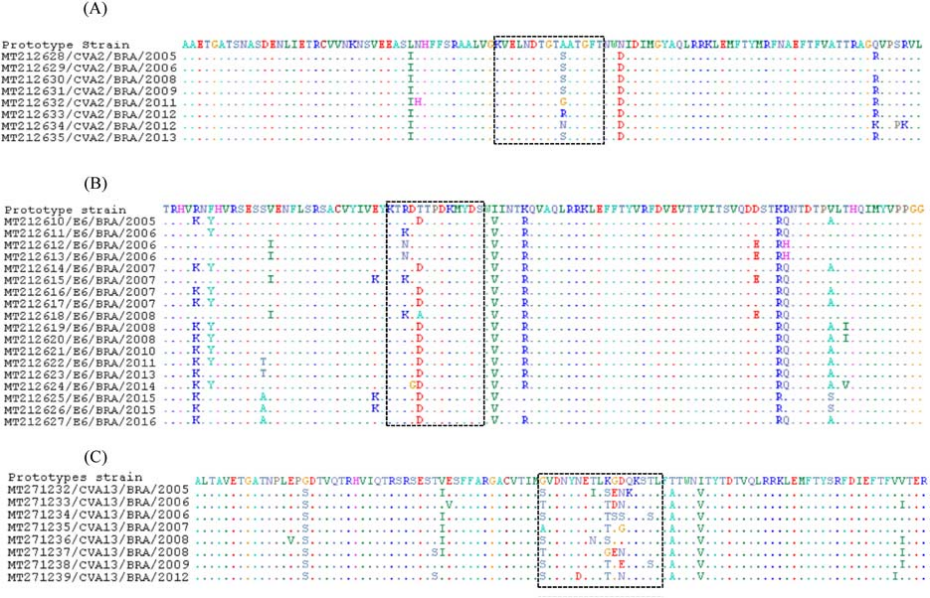
Deduced amino acid sequences of 100 residues in the BC loop of CVA2 (A), E6 (B) and CVA13 (C) VP1 sequences. The BC loop is highlighted in box. Aminoacid numbering was determined according to respective prototype strains AY421760 (CVA2), AY302558 (E6) and AF499637 (CVA13).

The VP1 region comprises major neutralizing antigenic sites, including the BC loop – an important immunogenic site located between the β-sheets B and C [26,27]. Thus, we explored the deduced amino acid sequences from the BC loop and surrounding regions of prototype strains and some viral types. Because length differences in VP1 were described in the amino terminal domain, the BC loop and carboxyl-terminal domain [28], we defined the VP1 BC-loop positions, as previously reported [29,30]. Among CVA2 isolates, the substitution A102S was found in almost all analysed sequences. Furthermore, three other sequences presented different substitutions in the same position Ala→Gly, Ala→Arg and Ala→Asn (Figure 3(A)). Among E6 isolates, five different substitutions were found. R82K and T84D were shared by Brazilian and representative sequences, whereas R82N, D83G, T84A were only observed in single Brazilian isolates (Figure 3(B)).

Many substitutions were found among CVA-13 isolates (Figure 3(C)). Aminoacids 168, 177 and 178 presented eight different substitution patterns: Gly→Ser, Gly→Thr, Gly→Ala; Lys→Ser, Lys→Thr, Lys→Gly and Gly→Glu, Gly→Asp, respectively. Four substitutions were detected in the position 179 (Asp→Asn, Asp→Ser, Asp→Gly and Asp→Glu). Further aminoacid replacements in the BC loop surrounding regions were perceived, especially in the E-6 and CVA-13 serotypes (Figure 3(C)).

To evaluate the probable effects of the aminoacid substitutions in the context of IgG–EV interaction, we carried out a molecular modelling analysis using our E6 isolates (supplementary Figure S2). Our findings suggested a small change in the net charge and volume with a limited effect of these substitutions in the BC loop region.

## Discussion

Since global wild poliovirus eradication is near to completion, the vast majority of the existing AFP cases have been associated to other etiologies where NPEVs play an important role [9, 11]. The Monitoring NPEVs circulation, such as EV-D68 and EV-A71, which also causes severe neurological disease [31] must be a key component of AFP surveillance, designed to control emergence/re-emergence of WPV and/or vaccine derived polioviruses (VDPV).

Studies on NPEVs based on poliovirus surveillance systems provided valuable information about circulation patterns and genetic diversity of these viruses, including the identification of novel endemic types [16,32,33]. Despite of this, although Brazil has accumulated enough information related to WPV and VDPV and the NPEVs isolation rate being satisfactory [12,34–36], little is known about the NPEVs epidemiology. This report addressed this question, providing data on the epidemiological patterns and genetic diversity of NPEVs identified in AFP cases (2005–2017).

In the present study, the NPEV isolation efficiency in different cell culture systems revealed a higher susceptibility to viral infection of RD (59.3%) compared to HEp-2C cells (15.1%). EV-A and EV-B species were more efficiently isolated in RD cells, in contrast to EV-C species, more promptly isolated in HEp-2C cells. Almost 15% of all NPEVs isolates and 60% of all NPEVs typed from HEp-2C cells were EV-C (EV-A:1.5% and EV-B:38.5%). In accordance to previous studies [37–39], these findings reinforce the need to include the HEp-2C cell culture in the current NPEVs surveillance protocols, in order to avoid EV-C underestimation.

Interestingly, it has been suggested that climatic factors, as humidity and elevated temperature, seem to favour EV transmission and isolation rates of NPEVs [40]. However, we did not observe clear seasonal distribution patterns, in line with previous observations. In fact, due to complexity of the diseases caused by enterovirus mainly in neurological manifestations it may be hard to explain the influence of climatic factors on enterovirus infections [41].

The present study revealed that EV-A, EV-B and EV-C species accounted for 29.3%, 57.5% and 13.2% of all isolates, respectively. The high prevalence of EV-B species and the detection pattern EV-B > EV-A > EV-C is aligned to other AFP surveillance reports [16,33,42], even though the prevalence of EV-C can be eventually higher than EV-A infections in some contexts [32,37,38]. Our results on the higher frequency of EV-B infections reinforce previous data on the increase of aseptic meningitis cases associated to EV-B infection in Brazil [43]. This work also identified a number of EV-A isolates, which was higher than EV-B/EV-C during the years 2011–2012. Furthermore, it is worth to mentioning that EV-C species was not more prominent than EV-A species in this study. This is the opposite of what was suggested for tropical countries [39], although in 2008 and 2017 the number of EV-C species isolates had been slightly higher. Moreover, we observed a slight fluctuation in the number of isolates that were typed as EV-B species, which decreased from 2010 to 2012 while the EV-A species increased in frequency. This pattern was reversed between 2012 and 2014 and the cycle looks to repeat itself in 2015. To the best of our knowledge, this is the first report on a putative fluctuation between EV-A and EV-B species associated with enteroviruses surveillance. Further studies are required to understand these temporal fluctuations.

Our findings also highlight that Echoviruses (n=141) and CV-A (n=105) were the major detected EVs among AFP patients. A high frequency of Echoviruses and CV-A has been described in many countries [16,32,33,37,42,44]. Although CV-A seems to be responsible for less than 1% of all EV-types associated with aseptic meningitis cases in Brazil [43], it was frequently found in association with AFP cases in this study. Altogether, these results suggest different circulation patterns of these viruses associated with AFP cases and other neurological disease in the country. Further studies must be conducted to ascertain regional features during EV infection.

According to previous studies, E6 and E11 have been the most commonly detected EV types, especially among patients showing severe central nervous system syndromes [16,30,33,42–44]. In this investigation, EV-A71 was the main pathogen associated with AFP (7.6%), followed by E6 (7.3%) and E11 (6.7%) suggesting a high occurrence of these types from 2005 to 2017. Indeed, the detection rate of these viruses observed here corroborates other studies conducted within AFP surveillance [33]. Although EV-A71 has been regularly detected in many studies involving AFP surveillance [16,33,37,42,44–46], it is not common to be the main EV type associated with AFP as demonstrated in the current study. Surprisingly, while EV-A71 point out as major EV type associated with AFP, a recent report suggested that it represented less than 1% of all types associated with aseptic meningitis in Brazil [43], whereas E6 played a relevant role in both neurological disorders, AFP and aseptic meningitis [43]. These findings may suggest a higher tropism to neuronal cells of E6 in comparison to EV-A71. In fact, EV-types present different cell/tissue tropisms, as a consequence of host and viral factors [47]. Further studies are needed to better understand the mechanisms and main factors associated to viral neurotropism.

Although an increasing number of HFMD and aseptic meningitis cases associated with CVA6/CVA16 and E30 infections, respectively, has been reported in Brazil [43, 48], an increase isolation rate of these EV-types was not observed among AFP patients. Consistently, even in countries with a high prevalence of these EVs, such as China and Brazil, these viral types seem to be poorly involved in AFP disease [43,48,49].

Another remarkable finding was the first description of some EV-types in Brazil, such as CVA2, CVA10 and EV-90. However, the identification of these EV-types does not mean a new or recent introduction into Brazil, but that they have not been previously identified. Similarly, this work also reports the detection of uncommon EV-types associated with AFP, such as CVA11, EV-90 and CVA12 [11].

Although E6, E11 and EV-A71 were the most detected EVs, it is worthwhile to highlight two other important types belonging to EV-C species, CVA13 and EV-C99. These types represented almost 10% of all EV identified in this study. These findings add valuable data on the role of these EV-types in neurological syndromes [11]. Interestingly, previous work have demonstrated that the co-circulation of a high number of different NPEV types, especially those from EV-C strains, favours recombination events [4-5] as shown by the recent isolation of type 2 VDPV polioviruses from the environment and the identification of an uncommon recombinant type3/type2 Sabin-related poliovirus in Brazil [34,35]. Moreover, multiple infections by different NPEVs have also been associated with atypical clinical presentations [7,50].

EV surveillance alongside with regular viral sequencing is relevant to (i) understand temporal and geographical patterns of viral dispersion; (ii) investigate putative associations between EV genotypes and clinical outcomes and (iii) timely identify the emergence of novel recombinant viruses of public health relevance [4,5,34,35,43,51–53]. This study adds valuable information on EV genetic diversity in Brazil, contributing to the knowledge on EV infections in South America, where data on viral sequencing is still scarce.

From 2006 to 2017, we observed the spread of three different E6 genogroups in Brazil, prevailing the GenIII in recent years. Despite periods of co-circulation, the replacement of genogroups along time was evident, reinforcing previous observations on the global [22] and Brazilian contexts [43]. Our data also document the genetic variability of E6 viruses, evidenced by the co-circulation of different clades from each genogroup. Furthermore, no clear temporal or geographical patterns were perceived, in line with previous reports [22].

CVA2 is detected worldwide and has been responsible for outbreaks [21,54]. Based on nucleotide sequences of the entire VP1 gene, CVA2 could be classified into four different genotypes and subgenotypes [20]. Brazilian CVA2 sequences belonged to genotype C (subgenotype C2) and closely clustered with a representative from Venezuela. Given the currently available global sequences, we did not find further close genetic relationships between our Brazilian sequences and those sampled outside of South American (supplementary Figure S1). Interestingly, among E30 Brazilian viruses, a close relationship between viruses circulating in Brazil, USA and South-American countries had been reported [43]. The USA representative from our study belonged to subgenotype C1, composed of sequences from different continents and assessed along a decade, proposing the global distribution of this subgenotype. Our study also corroborates findings of Yang et al. on the geographical restriction of genotype D infections within China [20]. CVA2 has played a major role in herpangina [54], which is not a notifiable disease in Brazil. Thus, CVA2 disease can be underestimated, as observed in other countries [20].

CVA13 VP1 sequences present a high genetic diversity and different viral clusters have been described (A-F) [17,55]. As little was known about the genetic variability of CVA13 viruses circulating in Brazil, we analysed sequences obtained from different geographical regions, assessed from 2005–2012. All Brazilian viruses belonged to cluster B and were distributed into two distinct clades. In contrast to CVA2 and E6, no relatedness between Brazilian viruses and those circulating in other South American countries was perceived. Noteworthy, Venezuelan viruses composed a new putative cluster, showing 72.5% to 75.8% of nucleotide similarity with former CVA13 clusters A to D [17]. Altogether, these figures suggest the circulation of multiple viral clusters in South America.

Our phylogenetic results suggest a diversity of E6, A71, CVB5 and E7 in Brazil, with circulation of distinct genotypes, genogroups or clades, in line with previous reports in the country [43] and elsewhere [22,38,56–60]. In addition to the lack of a clear geographical pattern of viral dissemination among the Brazilian regions, these figures suggest the occurrence of multiple introduction events. The apparent lower variability of CVA2 and CVA13 viruses in this sample – when compared to those other explored EVs – should be interpreted in the light of the small number of assessed sequences (n=8, each). For those presented as supplementary material, the same is true due to the small sequenced fragment (about 300 bp). Further analyses, comprising a more representative sample are necessary to compose a better picture of viral variability and circulation in the country. Besides the identification and characterization of novel viral types, the increasing number of deposited complete VP1 or full genome sequences will allow improvement of NPEV genotyping and a better understanding on the patterns of viral dissemination within and between countries and continents – a critical information to tailor global surveillance initiatives.

In this study, we also analysed the BC loop region, which is associated with viral antigenicity and can play a relevant role in host-virus interactions [26]. At least, three aminoacid substitutions within this loop and in adjacent regions were found in CVA2, E6 and CVA13 sequences. A higher variation was observed among CVA13 sequences, with potential impact on the three-dimensional protein structure. The putative impact of these mutations on viral antigenicity, fitness and virus-host cell interaction must be further ascertained.

In conclusion, the present study reveals the circulation of many different NPEVs types and genetic variants of these viruses in Brazil. Many of these EV-types have not been frequently associated with AFP, highlighting the need of studies on their role in the context of the central nervous system infections. Considering that EV infections are a public health concern, EV surveillance policies and strategies should be continuously tailored to allow the timely identification of novel emergent viruses and to improve monitoring of those already in circulation.

## Supplementary Material

Supplementary_figure_S2.docx

Supplementary_figure_S1.docx
