# Supplementary material for: Molecular characterization and epidemiological aspects of non-polio enteroviruses isolated from acute flaccid paralysis in Brazil: a historical series (2005–2017)
Source: Emerg Microbes Infect. 2020 Dec 1;9(1):2536–46. doi: 10.1080/22221751.2020.1850181 (PMC7717866; doi:10.1080/22221751.2020.1850181)
Supplement: Supplementary_figure_S2.docx [file TEMI_A_1850181_SM9665.docx]

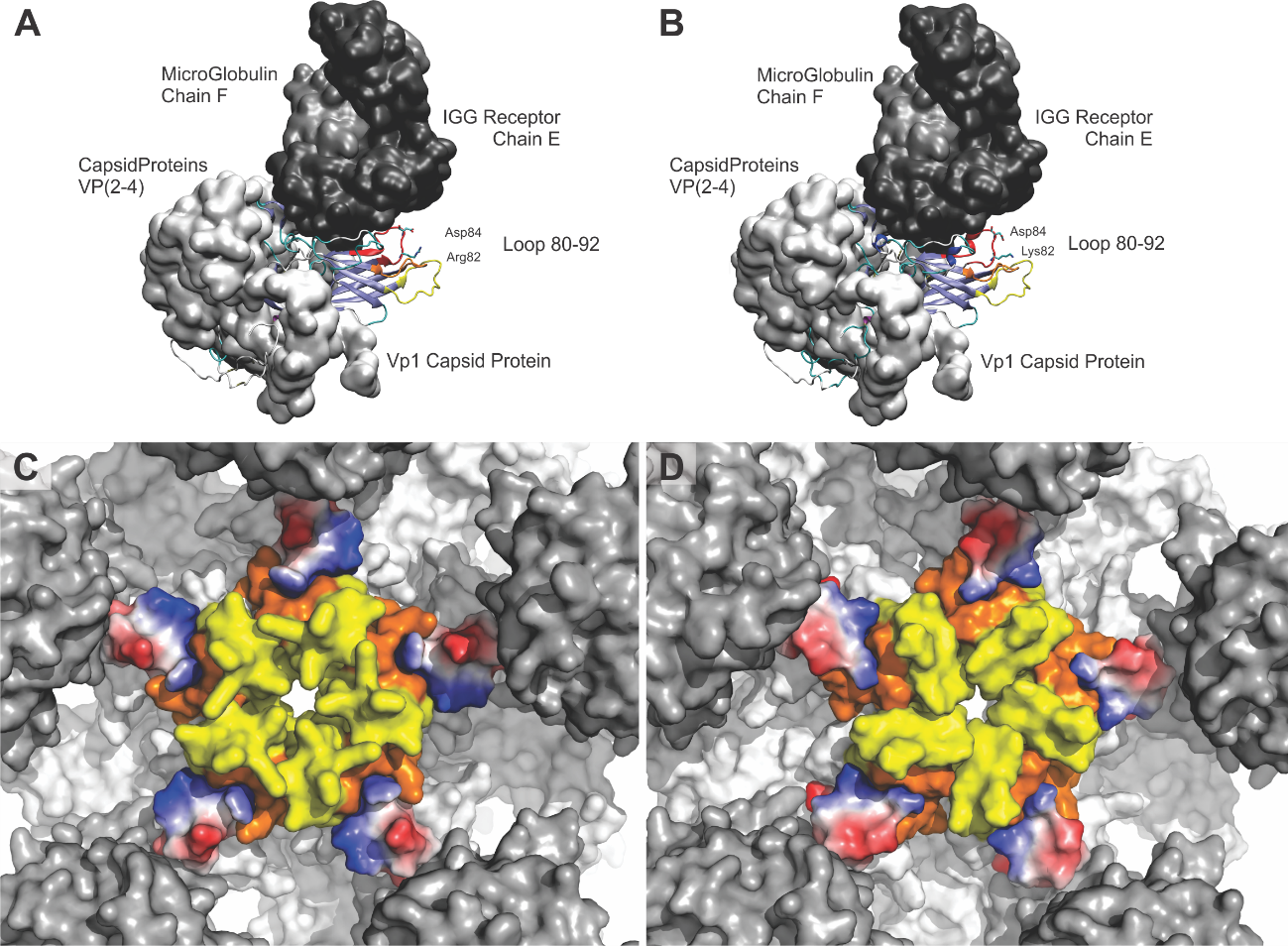


**Figure S2: Effect of Substitution R82K inside BC-loop in the Structure of E6 interacting with the IgG Receptor.** (A) Cryo-EM Structure of capsid proteins (VP1, VP2, VP3 and VP4; white surface) interacting with the IgG receptor (dark gray surface). VP1 was represented by cartoon diagram showing the BC loop (residues 80 to 92, in red) exposed in the capsid surface. (B) The model of VP1 R82K mutant showing limited effect on BC loop region. (C) Surface representation of a complete icosahedral face, following the same color scheme of A. BC-Loop is near the surface interaction with IgG, showing two opposite charges; one negative charge at carboxylic acid of Asp82 (red surface) and one positive charge at guanidino group of Arg 82, colored in blue. In (D) the Mutant R82K surface was represented showing a small change in the net charge and volume for BC loop.
