# Supplementary material for: Molecular characterization and epidemiological aspects of non-polio enteroviruses isolated from acute flaccid paralysis in Brazil: a historical series (2005–2017)
Source: Emerg Microbes Infect. 2020 Dec 1;9(1):2536–46. doi: 10.1080/22221751.2020.1850181 (PMC7717866; doi:10.1080/22221751.2020.1850181)
Supplement: Supplementary_figure_S1.docx [file TEMI_A_1850181_SM9664.docx]

A


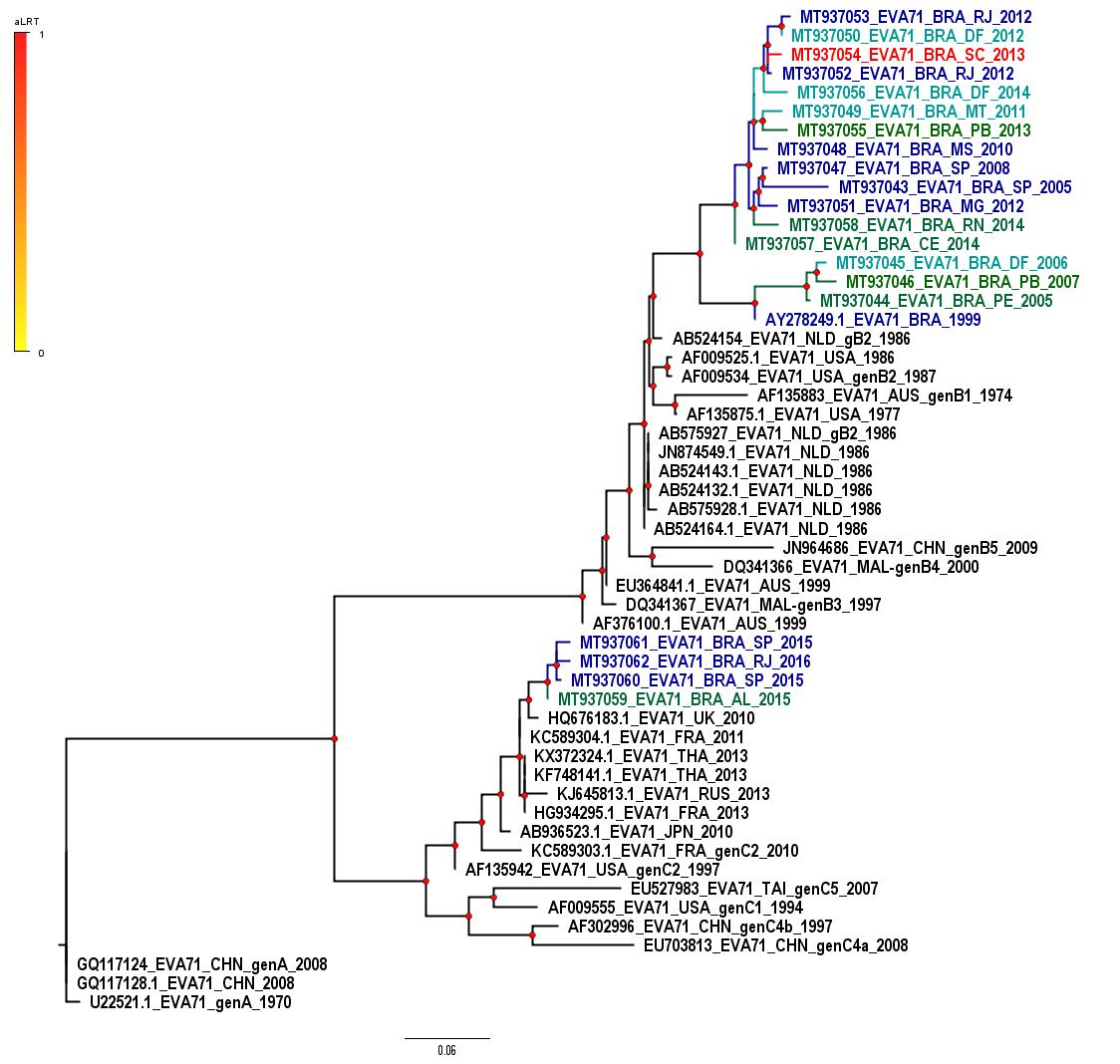


**Genotype A**

**Genotype C**

**Genotype B**

Genotype B2

Genotype C2

A


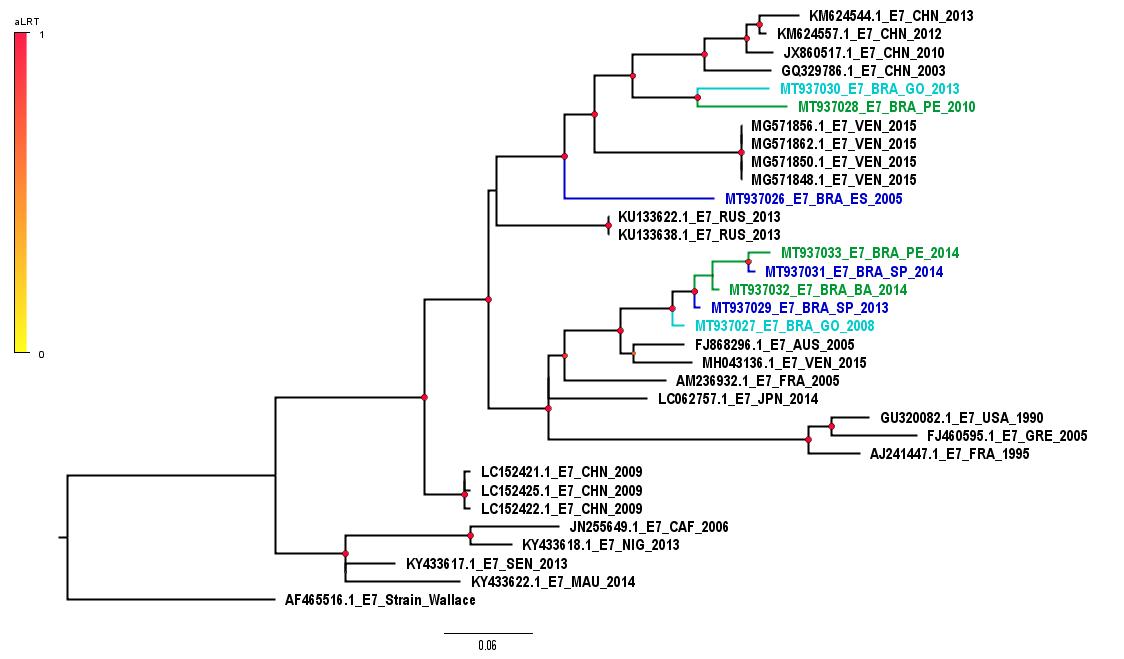


B

C


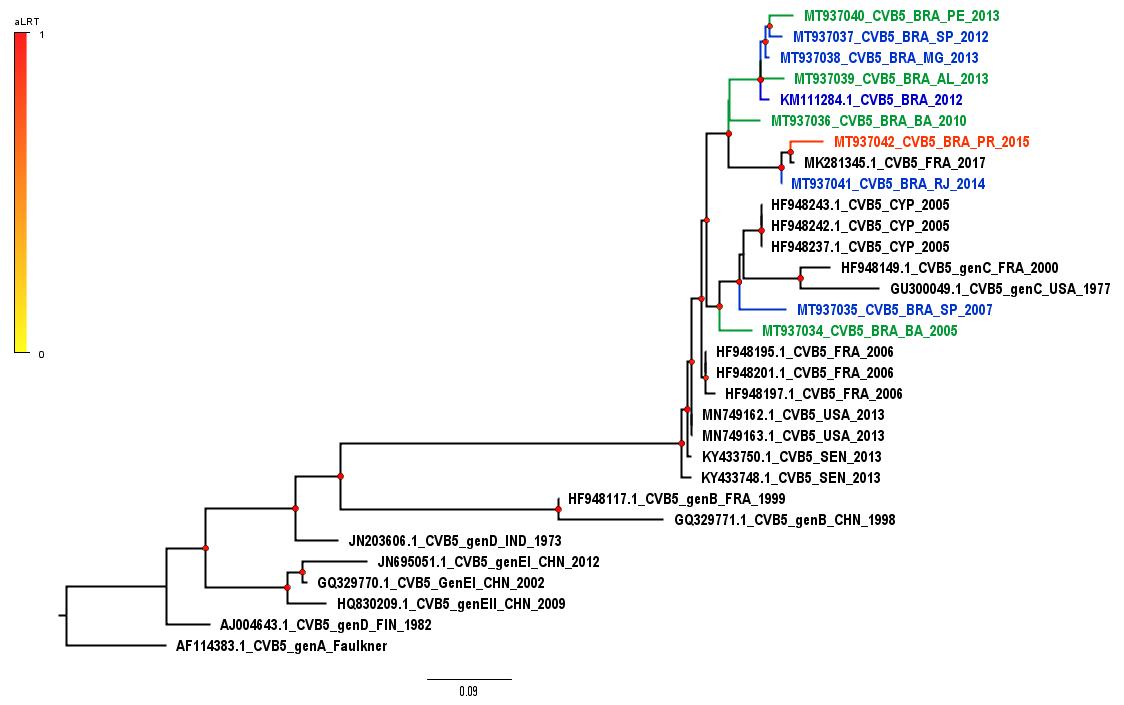


**Genogroup A**

**Genogroup D**

**Genogroup E**

**Genogroup B**

**Genogroup C**


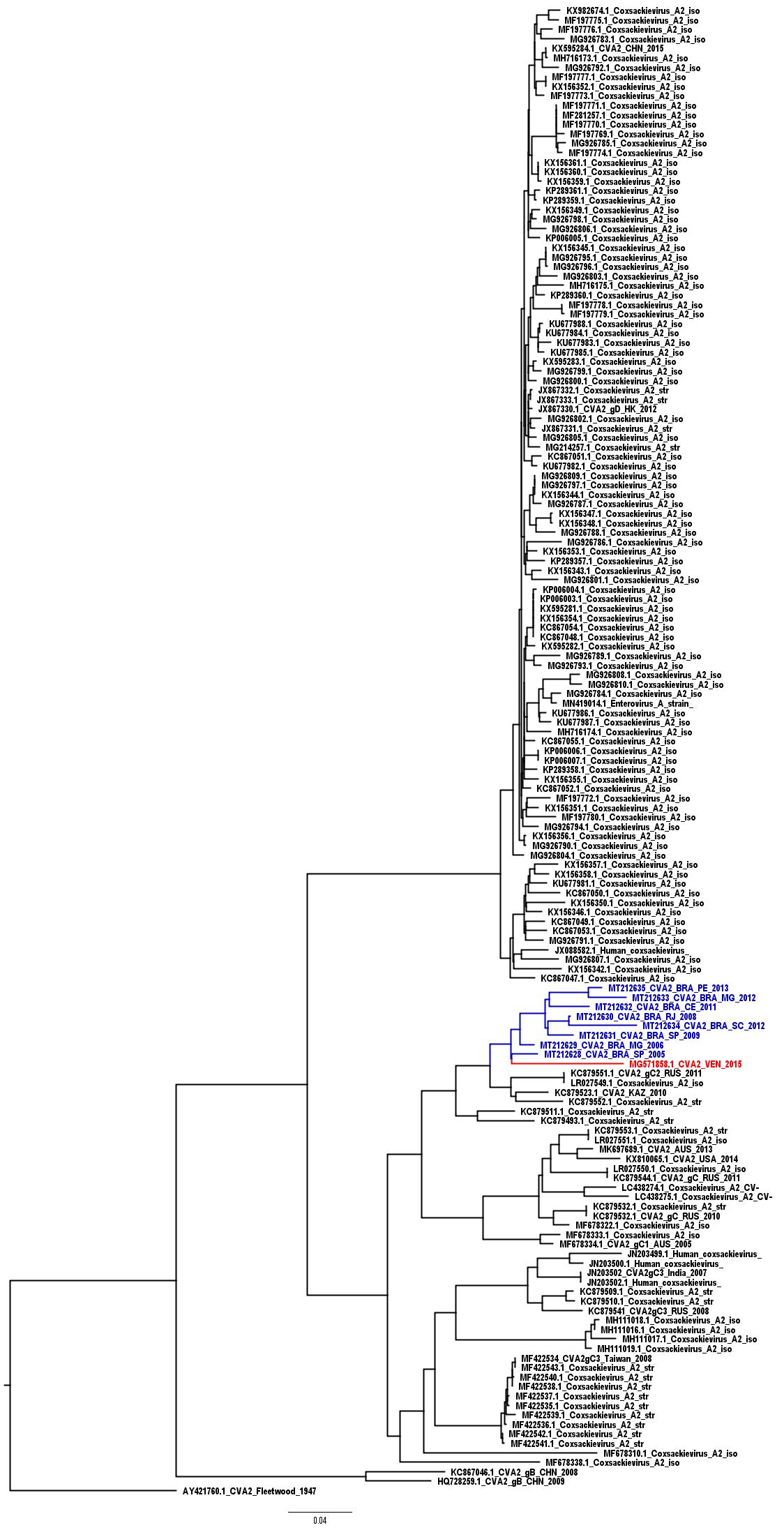


D

**Figure S1: Maximum likelihood phylogenetic trees of Brazilian and global representative based on VP1 sequences**. (A) EV-A71(300bp), (B) E7 (225bp), (C) CVB5 (221bp) and (D) CVA2 (869bp). The General Time Reversible model, with a discrete Gamma distribution and invariant evolutionary rate differences among sites was used as the nucleotide substitution model, as previously determined by J Model Test, version 2.1.4. CVB-5 genogroups were determined according to Liu et al., 2014. Sequences were colored according to the Brazilian geographical region: Northeast in green (AL, Alagoas; PB, Paraíba, PE, Pernambuco; BA, Bahia; CE, Ceará; RN, Rio Grande do Norte); Southeast in blue (ES, Espírito Santo; MG, Minas Gerais; RJ, Rio de Janeiro; SP, São Paulo); Midwest in light blue (DF, Distrito Federal; MT, Mato Grosso; GO, Goias) and South in red (SC, Santa Catarina; PR, Parana, RS, Rio Grande do Sul).
